# Omics analysis reveals the prognostic value of IPCDS models and potential targets for immunotherapy

**DOI:** 10.1007/s12672-026-04528-w

**Published:** 2026-01-27

**Authors:** Shenli Huang, Minmin Zhang, Yingjie Chen, Longgui Xie, Ziran Qiu, Na Jin, Wenqing Cao, Huawei Yang

**Affiliations:** 1 Department of Breast Surgery, Guangxi Medical University Cancer Hospital, Nanning, 530021 China; 2 Department of Breast and Thyroid Surgery, Liuzhou People’s Hospital affiliated to Guangxi Medical University, Liuzhou, 545006 Guangxi China; 3 Department of Emergency Medicine, Liuzhou Worker’s Hospital affiliated to Guangxi Medical University, Liuzhou, 545000 Guangxi China; 4 Department of Breast and Thyroid Surgery, Loudi Central Hospital, Hunan, 417000 China

**Keywords:** Breast cancer, Programmed cell death, Immune, Prognosis

## Abstract

**Background:**

Breast cancer remains a major threat to women’s health worldwide. The study aims to investigate the role of immune-related programmed cell death (IPCD) pathways and related genes in breast cancer progression and prognosis.

**Methods:**

We analyzed multi-omics datasets from TCGA, ICGC, and multiple GEO cohorts to screen for IPCD-related differentially expressed genes (DEGs) and established an IPCD-based signature (IPCDS) model for prognosis via 101 machine learning algorithm combinations. Functional enrichment analysis, survival analysis, principal component analysis (PCA), and immune correlation analysis were conducted by packages in R software.

**Results:**

The screened IPCD-related DEGs were primarily enriched in the MAPK and PI3K-AKT signaling pathways, as well as focal adhesion. Across different cohorts, patients in the high-IPCDS groups showed the worse overall survival than those in the low-IPCDS groups, indicating the robust prognostic value. The IPCDS model demonstrated a higher C-index than other published models. To predict the response to immunotherapy, application of the TIDE algorithm to TCGA data revealed a significant significant association between IPCDS and immunotherapy response; the low-IPCDS group had a lower TIDE score, while non-responders had a higher IPCDS score. Furthermore, the IPCDS score was negatively correlated with SIAH2 expression. High expression of SIAH2 predicted better survival and was inversely correlated with immune scores, suggesting its potential role as a protective biomarker [hazard ratios (HR) = 0.64]. A significant negative correlation was also observed between SIAH2 and CD8A expression (*p* = 0.0011).

**Conclusion:**

We established and validated a robust IPCDS model that effectively prognosticates breast cancer patients and predicts immunotherapy response. The model addresses the gap in integrating immune and programmed cell death pathways into a unified prognostic framework, outperforms existing models, and nominates SIAH2 as a potential key regulator. The elevated expression of SIAH2 is associated with a lower IPCD score, which in turn correlates with improved overall survival and reduced CD8 + T cell infiltration, highlighting its translational potential in guiding immune-targeted therapies.

**Supplementary Information:**

The online version contains supplementary material available at 10.1007/s12672-026-04528-w.

## Introduction

Breast cancer is the most common malignant tumor among women worldwide, with its incidence and mortality rates continuing to rise in recent years. According to statistics from the International Agency for Research on Cancer (IARC), breast cancer was the most commonly diagnosed cancer in women globally in 2022 [1]. Invasive breast cancer, as one of the most aggressive subtypes, is known for its high cell proliferation and invasive characteristics, often leading to a poor patient prognosis [2]. Due to the insidious nature of early symptoms, many patients are are diagnosed at advanced stages, having missed the optimal treatment window, which further exacerbates the threat of breast cancer to patients’ health [3]. Although traditional therapies, including surgical resection, chemotherapy, radiotherapy and endocrine therapy, remain the cornerstone of breast cancer treatment, these methods still face huge challenges in dealing with recurrence and metastasis [4]. Surgical resection, which aims to achieve a radical cure by removing the tumor tissue along with a margin of surrounding normal tissues, is a primary treatment modality. For patients with early-stage breast cancer, breast-conserving surgery combined with radiotherapy has become the standard of care, effectively balancing oncological outcomes with the preservation of breast appearance and function. However, surgical resection has limited efficacy in patients with advanced or metastatic breast cancer and is at high risk of recurrence after surgery [5]. For example, triple negative breast cancer (TNBC), as a highly aggressive subtype, lacks effective targeted treatment measures, and patient survival rates are significantly lower than other subtypes [6]. Therefore, finding more effective treatment strategies to improve patient outcomes represents an urgent unmet need for breast cancer research.

Advances in cancer immunology have established immunotherapy as a transformative approach in oncology. Immunotherapy activates the body’s immune system to mount a specific response against tumor cells, thereby achieving the goal of killing tumor cells [7]. In this context, the study of immune-related programmed death mechanisms is particularly important. Programmed death receptor 1 (PD-1) and its ligand (PD-L1) play an important role in regulating immune responses and maintaining immune homeostasis. However, tumor cells exploit the immunosuppressive function of the PD-1/PD-L1 signaling pathway to achieve immune escape, thereby promoting tumor occurrence and progression [8]. Studies have shown that blocking the PD-1/PD-L1 signaling pathway can effectively restore the anti-tumor activity of T cells, providing new ideas for breast cancer treatment [9]. Most existing prognostic models focus narrowly on either broad immune cell infiltration or a single programmed cell death pathway. Consequently, although genes related to immune checkpoints or apoptosis have been studied individually, their intricate crosstalk between immune function and diverse cell death mechanisms within the tumor microenvironment remains poorly represented in prognostic signatures. We hypothesize that an integrated focus on genes at the intersection of immune regulation and diverse programmed cell death pathways—termed immune-related programmed cell death (IPCD) genes—will yield a more robust and biologically coherent model. Thus, to address this gap, our study constructs an IPCD-based signature (IPCDS) to provide a more holistic and mechanistically informed prognostic tool for breast cancer. Research on immune-related programmed deaths not only helps to reveal the pathogenesis of breast cancer, but also lays a theoretical foundation for finding new therapeutic targets and optimizing existing treatment plans. Therefore, a deeper exploration of the role of IPCD in breast cancer holds substantial scientific and clinical value.

The aim of this study is to systematically analyze the impact of immune-related programmed death on breast cancer and its relationship with patient prognosis, thereby providing a theoretical basis for the optimization of clinical treatment options. Specifically, this study will focus on establishing an IPCDS for the prognosis of breast cancer, and evaluate its efficacy in predicting the survival rate and quality of life of patients. By integrating multi-center cohorts and employing multiple analysis techniques, this study seeks to overcome the limitations of existing studies, such as insufficient sample size and single analysis method, to yield more accurate and reliable results. The research results not only help to reveal the complex relationship between immune-related programmed death and breast cancer, but also provide an important reference for clinicians to formulating personalized treatment plans and prognostic assessments. Ultimately, this study is expected to contribute to improving the survival rate and quality of life of patients by promoting the optimization of breast cancer immunotherapy strategies.

## Materials and methods

### Transcriptome data acquisition

Transcriptome data was obtained from publicly available TCGA, GEO and ICGA databases to ensure comprehensive coverage and reliability of the study. The TCGA database provides high-throughput sequencing data, including RNA-sequencing (RNA-seq) data, from multiple cancer types, while ICGC focuses on characterizing the genomic alterations in various cancers across different populations. In this study, the TCGA-BRCA dataset (*n* = 1082) was downloaded, which includes transcriptome profiles of both tumor and normal breast tissue samples. The ICGC-BRCA dataset was also acquired for validation, which complements the TCGA data with additional patient samples and clinical information. The combined datasets for validation consisted of 4,374 samples from GSE1456 (*n* = 159), GSE20685 (*n* = 327), GSE21653 (*n* = 252), GSE3143 (*n* = 158), GSE42568 (*n* = 104), GSE58812 (*n* = 107), GSE9893 (*n* = 155), and brca_metabric (*n* = 1980), covering a diverse range of breast cancer chip data (Suppl. Figure 1 A). This comprehensive sample size enhances the representativeness and statistical power of the analysis, enabling robust identification of differentially expressed genes associated with breast cancer prognosis.

Totally 7 immunotherapy datasets were included for this study, referring to LUSC_GSE93157 (*N* = 13), Melanoma_GSE100797 (*N* = 25), Melanoma_GSE78220 (*N* = 28), Melanoma_GSE115821 (*N* = 33), Melanoma_PRJEB23709 (*N* = 91), Melanoma_phs000452 (*N* = 153) and nonsqNSCLC_GSE93157 (*N* = 22) in Suppl. Figure 1B-H. Differentially expressed genes (DEGs) between breast cancer samples and adjacent normal samples were identified based on TCGA (Normal = 113, Tumor = 1104) and GTEx (Normal = 179) datasets using the limma package in R, a widely used tool for analyzing microarray and RNA-seq data. The identified DEGs were further categorized based on their expression patterns, such as upregulated genes in tumor samples and downregulated genes in normal samples (Suppl. Figure 2), providing insights into the molecular alterations underlying breast cancer progression. The normalizeBetweenArrays function in the limma package was used to correct the chip data. The elimination of batch effects between data is performed using the Combat function in the sva package.

### scRNA-seq data processing

scRNA-seq data of 6 breast cancer tumor samples (CID3838, CID3921, CID4290A, CID4465, CID44971, and CID4535) from the GSE176078 dataset were analyzed using the Seurat package in R, following a standardized workflow for single-cell data processing. Quality control (QC) was performed to filter out low-quality cells and potential doublets based on standard metrics. Specifically, we applied the following thresholds: cells with fewer than 200 unique genes were considered potential empty droplets and were removed, while cells with more than 6,000 genes or with mitochondrial gene content exceeding 10% were flagged as potential doublets or cells with compromised viability, respectively, and were subsequently filtered out. Data normalization was then applied to account for differences in sequencing depth across cells, using the NormalizeData function in Seurat, which scales the expression values based on the total unique molecular identifier (UMI) count per cell. Dimensionality reduction was performed using the PCA method, followed by the identification of significant principal components (PCs) based on the elbow plot. Cell clustering was conducted using the FindClusters function in Seurat, which employs a graph-based algorithm to group cells with similar expression profiles. The resulting clusters were visualized using the Uniform Manifold Approximation and Projection (UMAP) technique, allowing for clear separation and identification of different cell types. This comprehensive processing pipeline ensures accurate characterization of the cellular composition and heterogeneity of breast cancer samples. The FindAllMarkers function was performed to calculate the DEGs between Cluster or cell types, with a threshold set at anadjusted p-value < 0.05, |log_2_FC| > 0.25, and the expression ratio should be greater than 0.1.

### Construction of the prognostic model

A prognostic model IPCDS based on IPCD genes [10]was constructed using 101 machine learning algorithm combinations for establish the risk score. The 101 combinations were generated by integrating 10 base machine learning algorithms, including Stochastic Survival Forest (RSF), Resilient Network (Enet), Lasso, Ridge, Stepwise Cox, CoxBoost, Cox Partial Least Squares Regression (plsRcox), Supervised Principal Component (SuperPC), Generalized Boosted Regression Model (GBM), and Survival Support Vector Machine (survival-SVM), with 10-fold cross-validation for robust parameter tuning and model training. This approach allowed us to systematically evaluate and compare the prognostic performance across a wide range of modeling strategies. The model with the highest average Harrell consistency index (C-index) across all validation datasets was selected as the optimal model. First, univariate Cox regression analysis was performed to screen IPCD genes that were significantly associated with overall survival (OS) in breast cancer patients (*p* < 0.01). The above machine learning algorithm model generates a risk score for each patient based on the expression levels of the selected IPCD genes and their respective coefficients, providing a quantitative assessment of the patient’s prognosis. The cutoff value of the group was determined according to the surv_cutpoint function, and all patients were divided into high-IPCDS group and low-IPCDS group. This approach allows for the identification of key IPCD genes that contribute most to the prediction of survival outcomes and enables the construction of a robust and interpretable prognostic model.

Survival curves were generated to compare the survival outcomes between high-risk and low-risk groups based on the risk score calculated by the model, with the log-rank test used to determine the statistical significance of the differences. Receiver operating characteristic (ROC) curves were constructed to evaluate the model’s ability to discriminate between patients with different survival outcomes, with the area under the curve (AUC) serving as a measure of prediction accuracy. The C-index, also known as the concordance index, quantifies the model’s ability to correctly rank patients based on their survival times, with higher values indicating better performance. For each model, the C-index is calculated in all validation datasets, and the model with the highest average C-index is considered the best. These evaluation metrics were applied to both the training set and the validation set to ensure the model’s reliability and generalizability. The results demonstrate that the model exhibits high accuracy and stability in predicting breast cancer prognosis, highlighting its potential value in clinical application.

### Cell-to-cell communication analysis

To explore potential cell-to-cell communication, we employed the CellChat package, a powerful tool designed for this purpose. Initially, we imported the normalized gene expression matrix, which formed the basis for creating a CellChat object. This object was then subjected to rigorous preprocessing using default parameters through the identifyOverExpressedGenes, identifyOverExpressedInteraction, and ProjectData functions. These steps helped to highlight genes and interactions that were significantly overexpressed. Subsequently, we performed a series of computations using the computeCommunProb, filterCommunication, and computeCommunProbPathway functions. These functions are crucial in identifying potential ligand-receptor interactions, which play a pivotal role in cell communication. Ligand-receptor interactions were prioritized based on their computed communication probabilities. Interactions with a probability < 0.01 were filtered out to focus on the most robust signals. The analysis included all known immune checkpoint pairs (e.g., PD-1/PD-L1, CTLA-4/CD80) present in the default CellChat database. The results of these analyses were further refined through the aggregateNet function, which generated a comprehensive cell communication network. This network provided valuable insights into the intricate interactions between cells in the context of the study.

### Mutation analysis

In parallel, we used GISTIC 2.0 software to analyze the copy number variations (CNVs) of each patient. CNVs are known to play a significant role in the development and progression of various diseases, including cancer. By analyzing these variations, we aimed to understand their potential impact on the disease state. Additionally, we calculated the tumor mutational burden (TMB) of each patient using maftools software. TMB is an important indicator of the extent of genomic instability in a tumor and has implications for therapeutic strategies and patient outcomes.

### Correlation analysis between prognostic models and tumor immunity

To ascertain the degree of immune infiltration in each patient from the TCGA database, we utilized IOBR software. This software contains the results of seven distinct assessment methods, each designed to evaluate different aspects of immune cell infiltration and activity. By applying these assessments, we aimed to gain a comprehensive understanding of the immune landscape within each patient’s tumor.

### Predicting response to immunotherapy

The Tumor Immune Dysfunction and Exclusion (TIDE) (harvard.edu) algorithm was used to predict the immune response in the TCGA dataset to evaluate the IPCDS differences in immunotherapy response.

### Statistical analysis

All data processing, statistical analysis and plotting are performed in R 4.1.3 software. The correlation between two continuous variables was assessed by the Pearson correlation coefficient. The chi-square test was used to compare the categorical variables, and the Wilcoxon rank-sum test was used to compare the continuous variables. The survminer package determined the optimal cut-off. Cox regression and Kaplan-Meier analysis were performed by the survival package.

## Results

### Discovering model genes

**Fig. 1:**
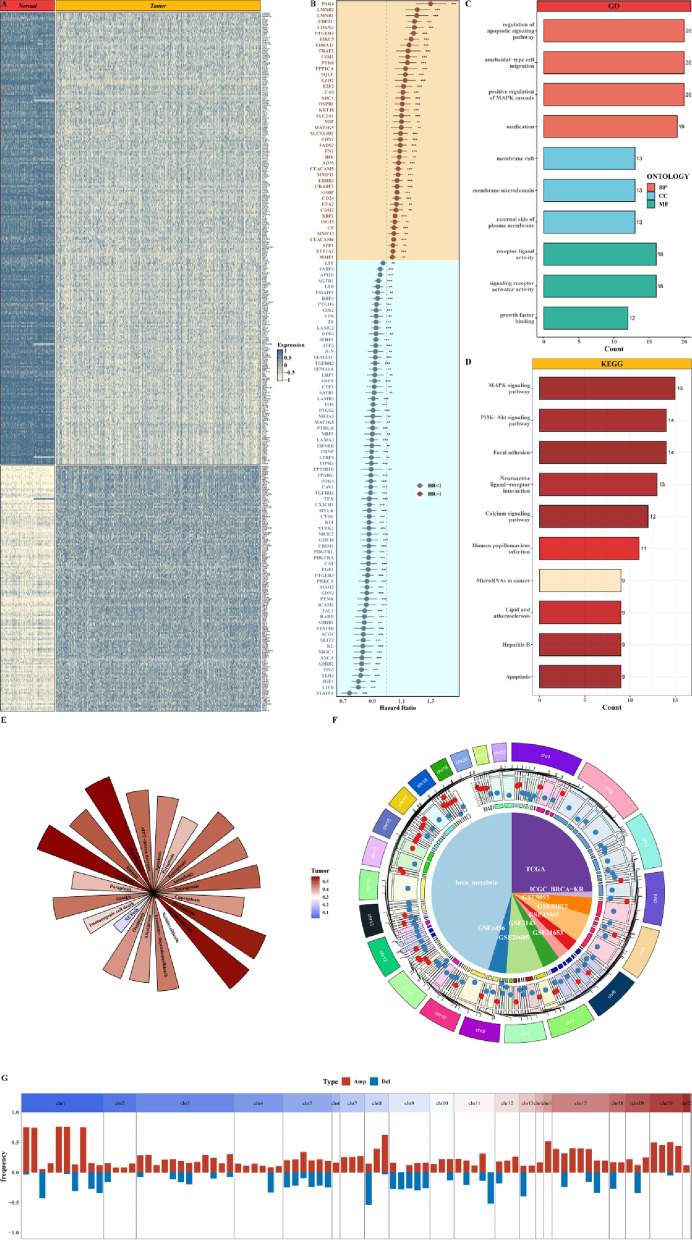
Screening and characterization of IPCD-related prognostic genes. **A** Heatmap of IPCD-related DEGs between tumor and adjacent normal samples, showing distinct clustering of upregulated (blue) and downregulated (pale yellow) genes. **B** Forest plot of the 118 prognostic IPCD genes, displaying HR and confidence intervals for each gene. **C**,** D** Histogram of significantly enriched GO terms and KEGG pathways among the prognostic IPCD genes, highlighting regulation of apoptotic signaling, MAPK cascade, and focal adhesion. **E** Histogram of ssGSEA scores for 20 programmed cell death pathways in TCGA breast cancer samples, revealing elevated activities of Disulfidptosis, Methuosis, Parthanatos, and Entotic cell death. **F** Circos plot mapping the chromosomal locations of the prognostic IPCD genes, illustrating their genome-wide distribution. **G** Bar plot showing the CNV frequency of prognostic IPCD genes, with red indicating amplification and blue indicating deletion

We performed limma package to calculate DEGs related to IPCD between tumor and paracancerous samples, showing distinct clustering of upregulated and downregulated genes (Fig. 1A). To further screen for clinically relevant IPCD genes, univariate Cox regression analysis was performed, which identified 118 prognostic genes, including protective genes and risk genes divided by hazard ratio as illustrated in the forest plot in Fig. 1B. They were mainly enriched for Gene Ontology (GO) terms of regulation of apoptotic signaling pathway, ameboidal-type cell migration, and positive regulation of MAPK cascade, and Kyoto Encyclopedia of Genes and Genomes (KEGG) pathways of MAPK, PI3K-AKT signaling pathways and focal adhesion (Fig. 1C-D). The Single-sample gene set enrichment analysis (ssGSEA) scores of 20 programmed cell death pathways in TCGA tumor data were shown, with higher scores of Disulfidptosis, Methuosis, Parthanatos, and Entotic cell death (Fig. 1E). The position of each prognostic IPCD genes from different datasets on the human chromosome was visualized in Fig. 1F, illustrating their genome-wide distribution. The CNV changes of each prognostic IPCD gene were exhibited in Fig. 1G and Suppl. Figure 3, with red indicating amplification and blue indicating deletion.

### Construction of IPCDS model

**Fig. 2:**
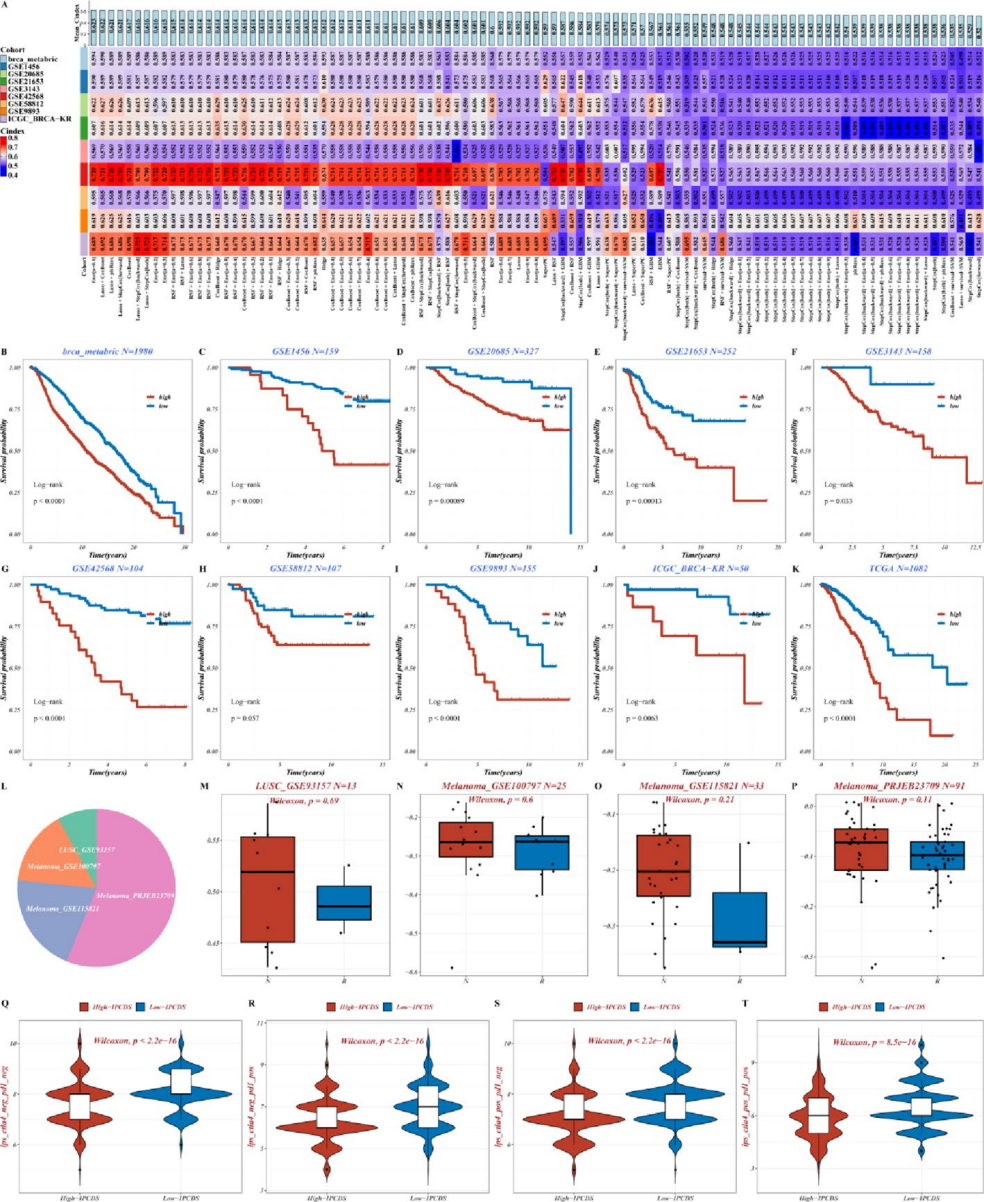
Construction and validation of the IPCDS prognostic model. **A** Heatmap of C-index values across 101 machine learning algorithm combinations, with the optimal model (Enet[α = 0.1]) ranking the first. **B–K** Kaplan-Meier survival curves for high- and low-IPCDS groups across multiple cohorts (TCGA, ICGC, and GEO datasets), demonstrating significantly worse overall survival in high-IPCDS patients (all log-rank *p* < 0.05 except GSE58812 dataset). **L–P** (Left) Pie chart of the composition of immunotherapy cohorts; (Right) Boxplots comparing IPCDS scores between these groups, showing higher scores in non-responders. **Q–T** Violin plots illustrating differences in IPS between high- and low-IPCDS groups across four IPS categories (IPS, IPS-CTLA4, IPS-PD1/PD-L1/PD-L2, and IPS-CTLA4 + PD1/PD-L1/PD-L2), indicating enhanced immunogenicity in the low-IPCDS group

We modeled the above DEGs using 101 algorithms, and the Enet algorithm with an alpha parameter of 0.1 (Enet[α = 0.1]) yielded the best performance (highest average C-index) and was thus chosen for constructing the final IPCDS model (Fig. 2A). Patients in the high-IPCDS group had a significantly poorer overall survival compared to the low-IPCDS group in the TCGA cohort (log-rank *p* < 0.0001), and this trend of significantly worse survival for the high-IPCDS group was consistently observed across all independent validation datasets (Fig. 2B-K, all log-rank *p* < 0.05 except GSE58812 dataset), robustly affirming the strong prognostic capacity of the IPCDS signature. Four immunotherapy data sets were used for verification, with the composition of them showed in pie chart (Fig. 2L), and the non-responsive sample IPCDS was greater than the responsive sample (Fig. 2M-P). Finally, immunophenoscore (IPS) values from the TCIA database were used to evaluate the differences in immunotherapy between high- and low-IPCDS groups across four IPS categories (IPS, IPS-CTLA4, IPS-PD1/PD-L1/PD-L2, and IPS-CTLA4 + PD1/PD-L1/PD-L2), indicating enhanced immunogenicity in the low-IPCDS group (Fig. 2Q-T).

### Model comparison

**Fig. 3:**
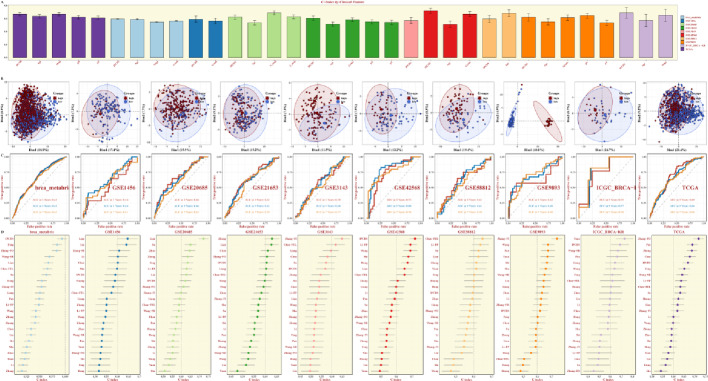
Comparative performance of the IPCDS model. **A** Bar charts comparing the C-index of IPCDS against traditional clinical variables (age, Stage, Grade) across all datasets, demonstrating superior prognostic performance of IPCDS. **B** PCA plots showing clear separation between high- and low-IPCDS groups based on the expression of model genes. **C** Time-dependent ROC curves evaluating the predictive accuracy of IPCDS for 3-, 5-, and 7-year overall survival across different cohorts. **D** Comparison of C-index values between the IPCDS and 22 previously published breast cancer prognostic models, showing IPCDS ranks among the top performers

The comparison the C-indexes of IPCDS against those of traditional clinical variables such as age, Stage, Grade, pM, pN, and pT for each dataset is shown in Fig. 3A, demonstrating superior prognostic performance of IPCDS. The PCA plots plotted based on the expression of model genes demonstrated clear separation between high- and low-IPCDS groups, and the time-dependent ROC (timeROC) curves for each breast cancer dataset were then presented, showing the great predictive efficacy via AUC at 3, 5, 7 years (Fig. 3B-C). To evaluate the prognostic efficacy of IPCDS against existing breast cancer prognostic models, we integrated findings reported in 22 other previous literatures that utilized biologically significant features, such as apoptosis, autophagy, m6A modification, necroptosis, the ubiquitin-proteasome system, inflammatory signatures, and immune checkpoints. Notably, the C-index of IPCDS ranked consistently among the top across all datasets, particularly in the brca_metabric, GSE42568, and ICGC-BRCA datasets (Fig. 3D).

### Tumor immunoassays

**Fig. 4:**
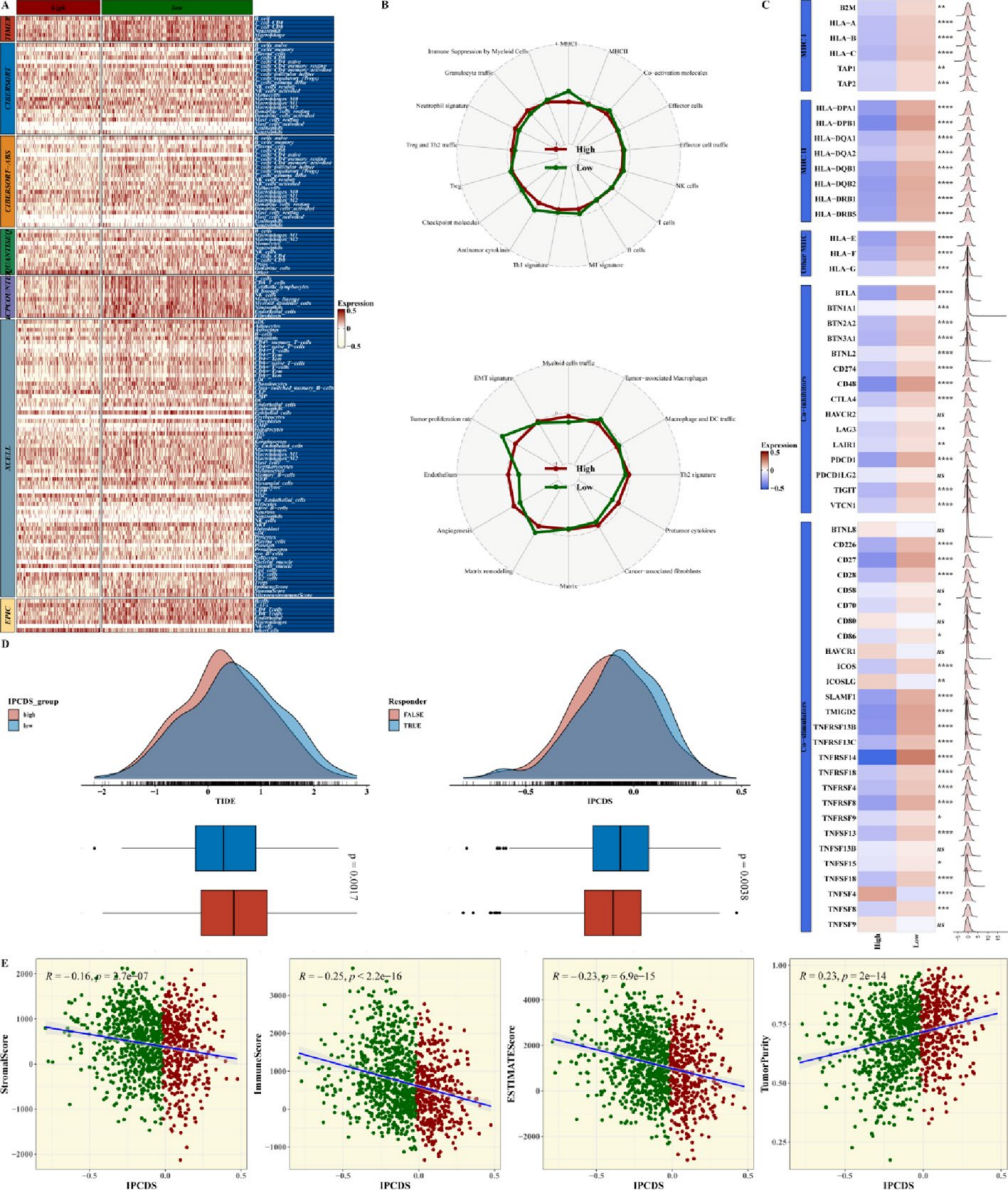
Association between IPCDS and tumor immune microenvironment. **A** Heatmap summarizing immune cell infiltration scores estimated by seven algorithms (including TIMER, CIBERSORT, EPIC, and so on), revealing significant differences between high- and low-IPCDS groups. **B** Radar chart illustrating of differences between IPCDS and immune-related pathways. **C** Heatmap displaying correlations between IPCDS and expression of immune-related genes. **D** (Upper) Probability density distributions of IPCDS scores for predicted immunotherapy responders and non-responders by TIDE algorithm; (Lower) Boxplot confirming significantly higher IPCDS in non-responders. **E** Scatter plots showing significant negative correlations between IPCDS and StromalScore, ImmuneScore, ESTIMATEScore, and positive correlation with TumorPurity (all *p* < 0.01)

The estimated results of the seven algorithms (including TIMER, CIBERSORT, EPIC, and so on) for assessing immune infiltration scores revealed significant differences between high- and low-IPCDS groups (Fig. 4A, Suppl. Figure 4). The radar chart of the differences of IPCDS with immune-related pathways in the high- and low-IPCDS groups was visualized in Fig. 4B. We then plotted a heatmap to present the expression of immune-related genes in the high- and low-IPCDS groups and their excellent correlation with IPCDS (Fig. 4C). The TIDE algorithm was conducted to the TCGA cohort to predicted immunotherapy response. The probability density distributions of IPCDS scores for predicted immunotherapy responders and non-responders demonstrated a significant difference between the two groups, with the low-IPCDS group having a lower TIDE value and the non-responders exhibiting significantly higher IPCDS scores than responders (Fig. 4D). The significant negative correlations between IPCDS and StromalScore, ImmuneScore, ESTIMATEScore, and positive correlation with TumorPurity predicted by ESTIMATE algorithms were demonstrated in Fig. 4E (all *p* < 0.01).

### Analysis of model gene SIAH2

**Fig. 5:**
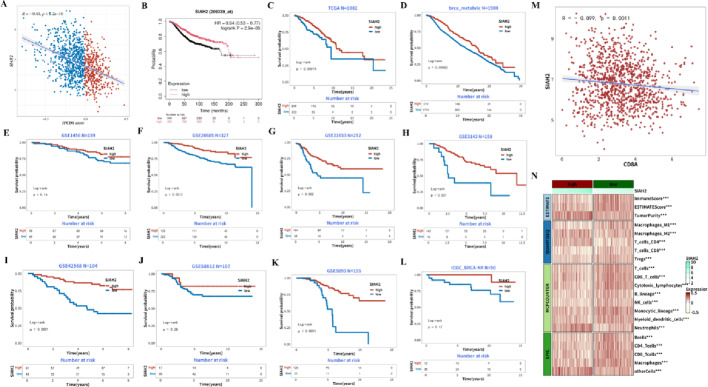
The analysis on the prognostic and immunomodulatory role of SIAH2. **A** Correlation scatter plot demonstrating significant negative correlation between IPCDS and SIAH2 expression (*p* < 0.01). **B** Kaplan-Meier survival analysis showing significantly better overall survival for patients with high SIAH2 expression. **C–L** Validation of SIAH2 prognostic effect across multiple cohorts (TCGA, ICGC, and GEO datasets), consistently showing improved survival with high SIAH2 expression. **M** Scatter plot revealing significant negative correlation between SIAH2 and CD8A expression (*p* = 0.0011). **N** Heatmap displaying correlations between SIAH2 expression and levels of various immune-infiltrated cells, suggesting its broad immunomodulatory potential

To analyse the the prognostic and immunomodulatory role of SIAH2, we conducted correlation analyses, survival analyses, immune-related analyses. The correlation scatter plot demonstrated that the IPCDS score was negatively correlated with the expression level of SIAH2 (Fig. 5A, *p* < 0.01). The Kaplan-Meier survival curves showed that patients with high-expressed SIAH2 levels have better survival probability than those with low-expressed SIAH2, serving as a potential protective biomarker (Fig. 5B). SIAH2 had a significant prognostic effect in each cohort with the low SIAH2 expression group having shorter survival time than the high SIAH2 expression group (Fig. 5C-L). Then we investgated the relationship between SIAH2 expression and immune genes, and found a significant negative correlation between SIAH2 level and CD8A expression (Fig. 5M, *p* = 0.0011). Heatmap of correlation between SIAH2 expression and levels of various immune-infiltrated cells was shown in Fig. 5N and Suppl. Figure 5, suggesting its broad immunomodulatory potential.

## Discussion

In recent years, significant progress has been made in the research on the relationship between immune-related programmed cell death and breast cancer at home and abroad, spanning fields from basic research and clinical trials. Early research focused on exploring the expression pattern of PD-1/PD-L1 in breast cancer tissues and its relationship with clinicopathological features. For example, it has been reported that PD-L1 expression levels are higher in patients with TNBC and show a positive correlation with the enrichment of tumor-infiltrating lymphocytes (TILs), suggesting that PD-L1 may be an important target for TNBC immunotherapy [11]. Subsequently, multiple clinical trials have further validated the effectiveness of immune checkpoint inhibitors in the treatment of breast cancer. For example, the IMpassion130 trial showed that patients with PD-L1-positive advanced TNBC who received PD-1 inhibitors in combination with chemotherapy experienced a significant increase in progression-free survival (PFS) [12].

However, conclusions regarding the effect of immune-related programmed cell death on breast cancer vary across studies, potentially due to differences in study methodologies, sample sources, and patient population characteristics. Notably, while some studies have used immunohistochemistry to assess PD-L1 expression, results are often less comparable due to technical variations in antibody selection and staining scoring criteria [13]. In addition, response rates to immunotherapy vary significantly across breast cancer subtypes. Immunotherapy is generally less effective in hormone receptor-positive breast cancer than in TNBC, likely due to reduced immune cell infiltration within the tumor microenvironment [14]. Another study found that combining immune cell infiltration density with PD-L1 expression levels influences the prognosis of breast cancer patients, suggesting the importance of integrating multiple immune markers [9]. These studies provide an important reference for further optimizing the immunotherapy strategy for breast cancer.

Although numerous studies have explored the role of immune-related programmed cell death in breast cancer, there are still some gaps or controversies that need to be addressed. First, most existing studies have focused on analyzing single immune markers (e.g., PD-L1), while paid little attention to their interaction with other immune molecules and the combined impact on prognosis. Second, many studies have been limited by small sample sizes and single-center data, which can introduce substantial bias and limit generalizability to real-world clinical settings. Furthermore, conclusions regarding the relationship between immune-related programmed cell death and breast cancer prognosis remain inconsistent, especially for patients with different subtypes of breast cancer, and the long-term survival benefit still needs to be further validated.

To address these limitations, our study employed a multi-center, large-sample design and multi-dimensional data analysis to investigate the impact and prognostic value of immune-related programmed cell death in breast cancer. Specifically, we integrated gene expression profiles, clinicopathologic characteristics, and long-term follow-up data to construct the IPCDS prognostic model, revealing the independent prognostic significance of IPCD markers in breast cancer. The results demonstrate that our model was compared with 22 existing biomarkers and signatures, indicating that IPCDS provides a more stable and mechanistically grounded assessment of the tumor immune context. The superior performance of IPCDS likely stems from its comprehensive basis in the biological crosstalk between immune surveillance and diverse programmed cell death pathways. This integrated approach captures a more integral aspect of the tumor microenvironment’s functional state compared to models based on single pathways or general proliferation markers, thereby offering a more robust and biologically contextualized prediction. Overall, these findings support the view that IPCDS serves as a more effective prognostic model for breast cancer. Furthermore,, this study explores the potential advantages of immunotherapy combined with traditional treatment models, and provide a scientific basis for the formulation of personalized treatment plans, thereby addressing translational gaps in previous research. Our TIDE analysis indicates that patients in the low-IPCDS group are more likely to respond to immunotherapy. The negative correlation between IPCDS and TIDE, allong with its positive correlation with cytolytic activity scores, suggests that a low IPCDS score reflects an immunologically ‘hot’ tumor microenvironment, characterized by effective T cell infiltration and function, thereby explaining its association with a higher likelihood of response to immunotherapy. This finding highlights the potential clinical applicability of the IPCDS score, suggesting that it could serve as a valuable non-invasive biomarker for identifying breast cancer patients who would benefit most from immune checkpoint inhibitors. This could potentially spare non-responders from unnecessary treatments and associated side effects.

In addition, this study identified SIAH2 as a key downstream factor associated with favorable prognosis for breast cancer. As a ubiquitin E3 ligase, SIAH2 promotes proteasome-dependent degradation of proteins through ubiquitination [15]. In our analysis, lower SIAH2 expression was significantly correlated with higher IPCDS scores and poorer response to immunotherapy, suggesting its role in maintaining an immunocompetent tumor microenvironment. SIAH2 showed significant prognostic value across all cohorts, with the high SIAH2 expression group exhibiting significantly longer survival time than the low SIAH2 expression group (*p* < 0.05). This potential protective function is consistent with previous findings: the consistent association between low SIAH2 expression in high-risk patients and poor survival across multiple cohorts, including TCGA and ICGC datasets, reinforces its role as a favorable prognostic biomarker. Notably, Bian et al. also reported immune dysregulation and upregulation of immune checkpoint molecules in high-risk patients. This overexpression is often associated with mechanisms of immune escape. However, Other reports indicate that SIAH2 enhances chemosensitivity and suppress tumor stemness in TNBC by modulating the Hippo pathway [16], and its expression is predominantly observed in estrogen receptor-positive tumors, where it may be upregulated via estrogen-mediated transcriptional responses [17, 18]. Given this complexity, the exact mechanisms by which SIAH2 influences breast cancer progression and immunity remain to be fully elucidated through targeted functional studies. Additionally, the negative correlation we observed between SIAH2 and CD8A expression indicates its potential effect on CD8 + T cell function and infiltration, which warrants further investigation into its immunomodulatory mechanisms. These findings position SIAH2 not only as a likely prognostic indicator but also as a potential predictor of immunotherapy efficacy, providing new perspectives for a deeper understanding of the pathogenesis of breast cancer and laying a theoretical foundation for optimizing immunotherapy strategies.

Although our study provides a robust bioinformatic framework, several limitations must be acknowledged. Firstly, the reliance on retrospective public datasets introduces potential biases from batch effects and population heterogeneity, which potentially limit generalizability. Future prospective studies in uniformly processed cohorts are warranted. Second, the clinical applicability of our model is constrained by two factors: limited long-term follow-up data and the computational nature of immunotherapy response prediction, which relies on algorithms like TIDE. Validation in real-world clinical trials is crucial. Finally, the study lacks experimental validation; the biological roles of IPCDS and candidate genes like SIAH2 are inferred from bioinformatic correlations. Direct functional experiments, such as gene knockdown or overexpression in cell lines and animal models, are required to establish causality and elucidate the underlying biology.

## Supplementary Information

Below is the link to the electronic supplementary material.

Supplementary Material 1: Figure 1 A PCA plots demonstrating effective batch effect correction across TCGA and other BRCA datasets. B. Pie chart summarizing sample distribution across immunotherapy cohorts. C-H. Kaplan-Meier survival analyses of IPCDS in independent immunotherapy cohort, validating its prognostic value in immunotherapy contexts.

Supplementary Material 2: Figure 2 Expression heatmap of the IPCD-related DEGs in the TCGA dataset, showing distinct clustering of upregulated (blue) and downregulated (pale yellow) genes.

Supplementary Material 3: Figure 3 A-B. CNV segment profiles of TCGA dataset generated by GISTIC2.0 software. C. The mutation results calculated by maftools software based on TCGA mutation data, and the CNV part is the CNV results run by gistic2.0 software, which are merged and visualized using ComplexHeatmap. D-G. Differences in broad and focal CNV Gain or Loss alterations between high- and low-IPCDS groups. H-I. Correlation between IPCDS and TMB in TCGA cohort. J. Survival analysis combining TMB and IPCDS stratification.

Supplementary Material 4: Figure 4 A Bubble plot of cell type-specific marker expression. B. t-SNE visualization of cell clustering and annotation results. C. t-SNE plots showing expression patterns of eight modeled genes. D. Bar plot comparing cellular composition between high- and low-IPCDS groups. E-F. Differential cell-cell communication patterns between high- and low-IPCDS groups. G. Communication network diagram of cell-cell communication in high- and low-IPCDS groups. H-I. Pathway-specific communication differences between high- and low-IPCDS groups in single-cell data. J. Scatterplot of communication strength differences between groups with high and low IPCDS in single-cell data.

Supplementary Material 5: Figure 5 A. Correlation heatmap between SIAH2 and immune checkpoint genes. B. Correlation analysis between SIAH2 and 50 HALLMARK pathways plus TIP (IMMUNO-ONCOLOGY) scores.

## Data Availability

The datasets generated during the current study are available from the corresponding author on reasonable request.
