# Supplementary figures and images for: Omics analysis reveals the prognostic value of IPCDS models and potential targets for immunotherapy
Source: Discov Oncol. 2026 Jan 27;17:337. doi: 10.1007/s12672-026-04528-w (PMC12917040; doi:10.1007/s12672-026-04528-w)

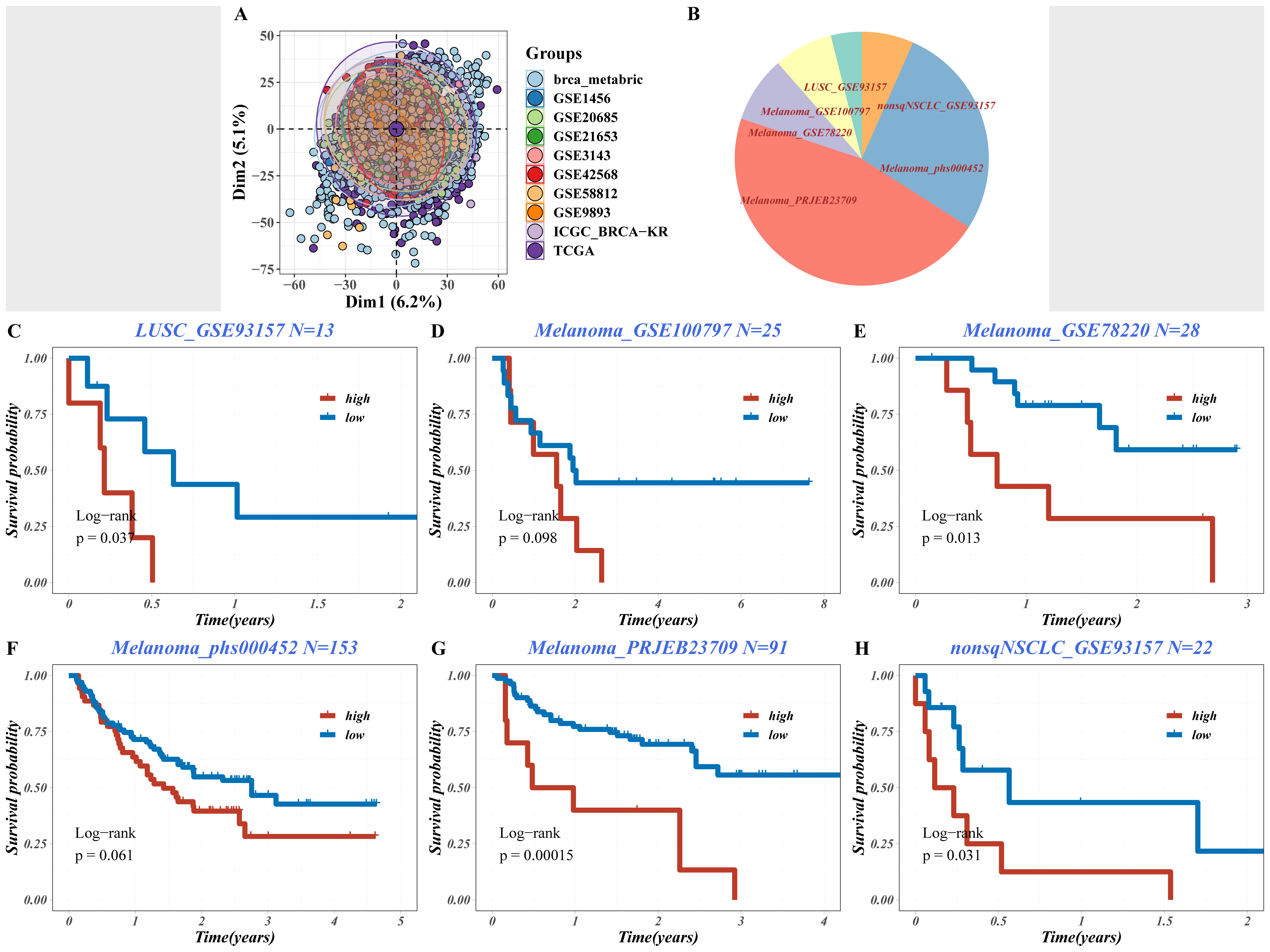

Supplement: Supplementary file 1 — Supplementary Material 1: Figure 1 A PCA plots demonstrating effective batch effect correction across TCGA and other BRCA datasets. B. Pie chart summarizing sample distribution across immunotherapy cohorts. C-H. Kaplan-Meier survival analyses of IPCDS in independent immunotherapy cohort, validating its prognostic value in immunotherapy contexts. [file 12672_2026_4528_MOESM1_ESM.tif]

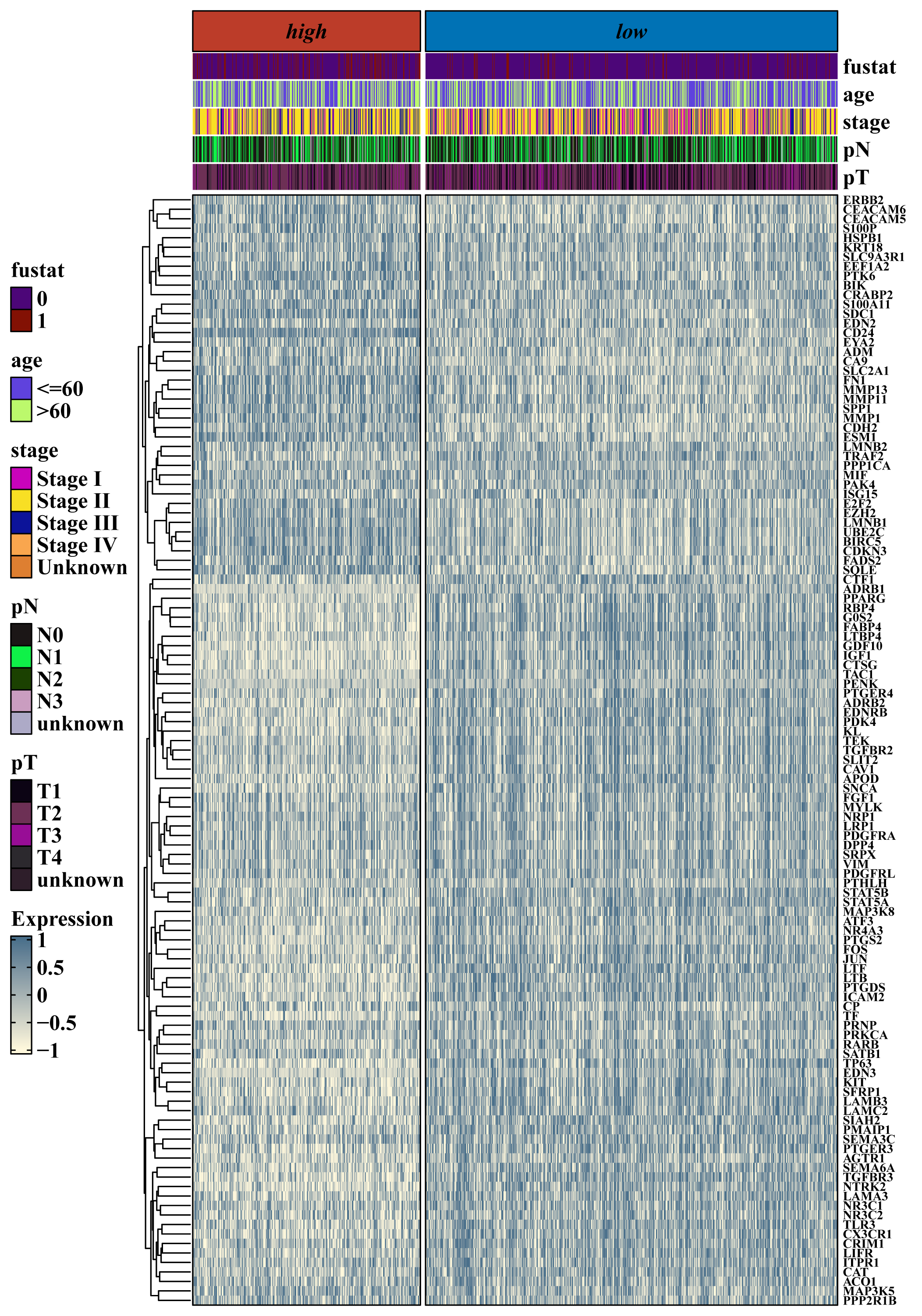

Supplement: Supplementary file 2 — Supplementary Material 2: Figure 2 Expression heatmap of the IPCD-related DEGs in the TCGA dataset, showing distinct clustering of upregulated (blue) and downregulated (pale yellow) genes. [file 12672_2026_4528_MOESM2_ESM.tif]

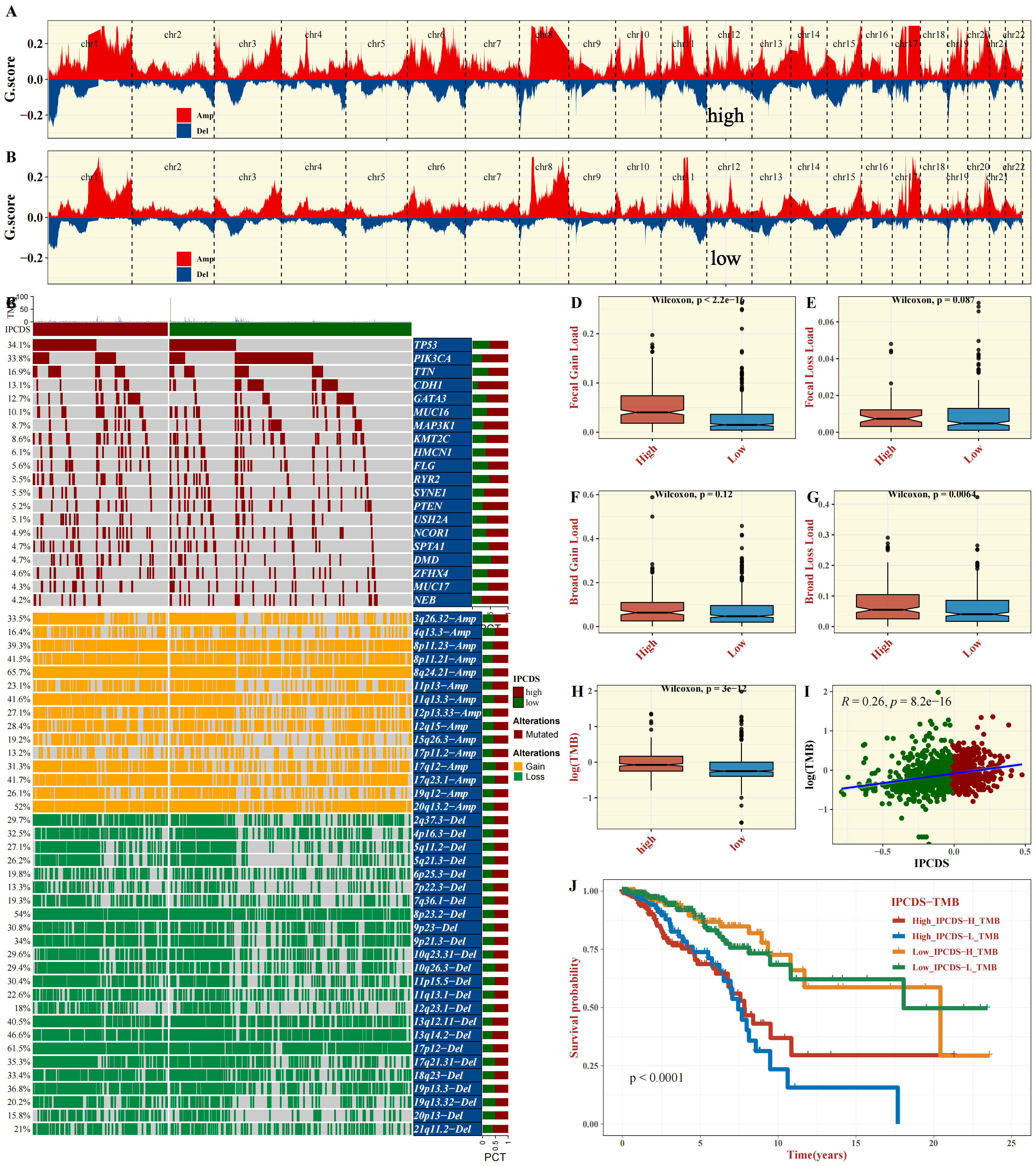

Supplement: Supplementary file 3 — Supplementary Material 3: Figure 3 A-B. CNV segment profiles of TCGA dataset generated by GISTIC2.0 software. C. The mutation results calculated by maftools software based on TCGA mutation data, and the CNV part is the CNV results run by gistic2.0 software, which are merged and visualized using ComplexHeatmap. D-G. Differences in broad and focal CNV Gain or Loss alterations between high- and low-IPCDS groups. H-I. Correlation between IPCDS and TMB in TCGA cohort. J. Survival analysis combining TMB and IPCDS stratification. [file 12672_2026_4528_MOESM3_ESM.tif]

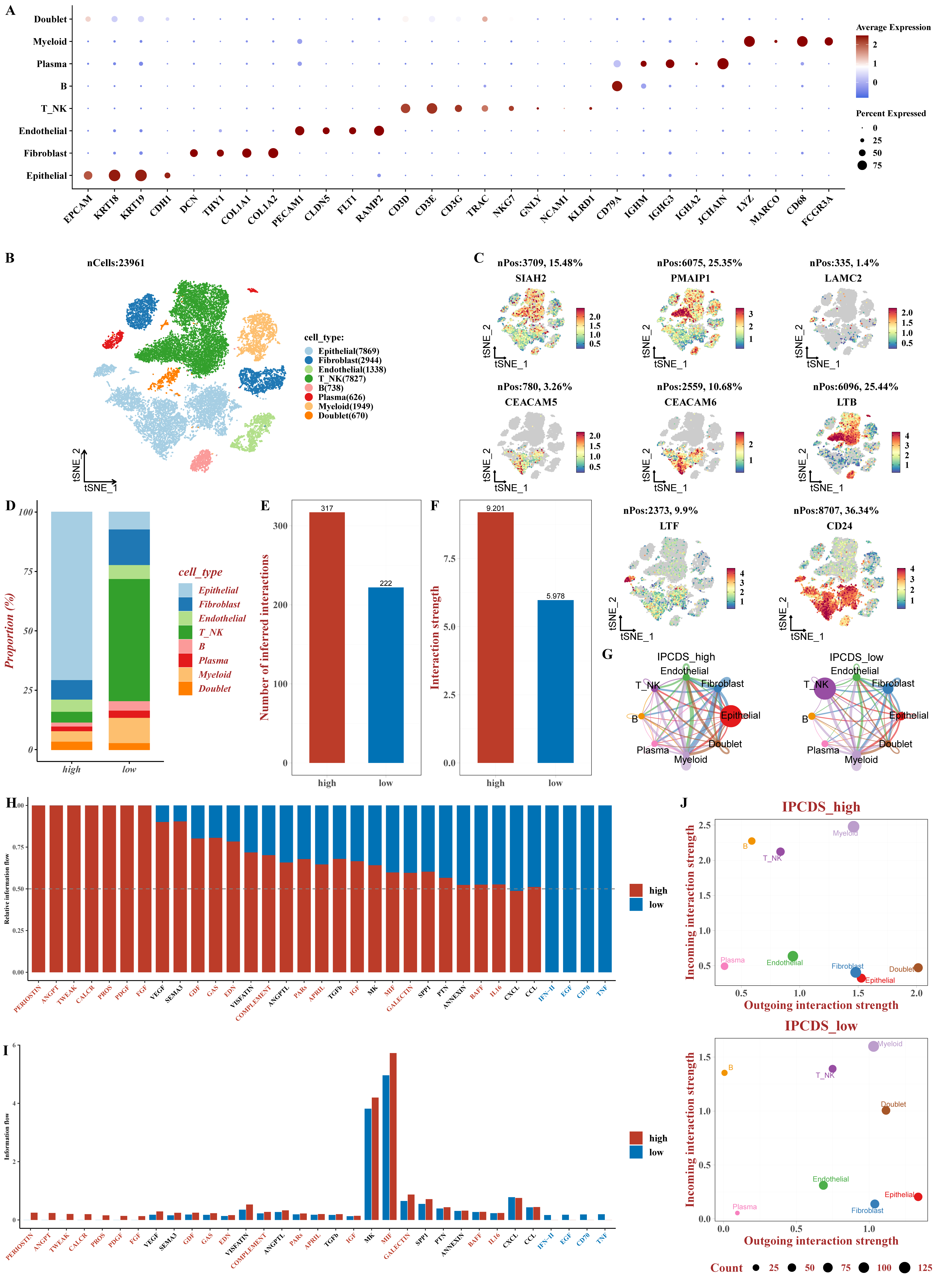

Supplement: Supplementary file 4 — Supplementary Material 4: Figure 4 A Bubble plot of cell type-specific marker expression. B. t-SNE visualization of cell clustering and annotation results. C. t-SNE plots showing expression patterns of eight modeled genes. D. Bar plot comparing cellular composition between high- and low-IPCDS groups. E-F. Differential cell-cell communication patterns between high- and low-IPCDS groups. G. Communication network diagram of cell-cell communication in high- and low-IPCDS groups. H-I. Pathway-specific communication differences between high- and low-IPCDS groups in single-cell data. J. Scatterplot of communication strength differences between groups with high and low IPCDS in single-cell data. [file 12672_2026_4528_MOESM4_ESM.tif]

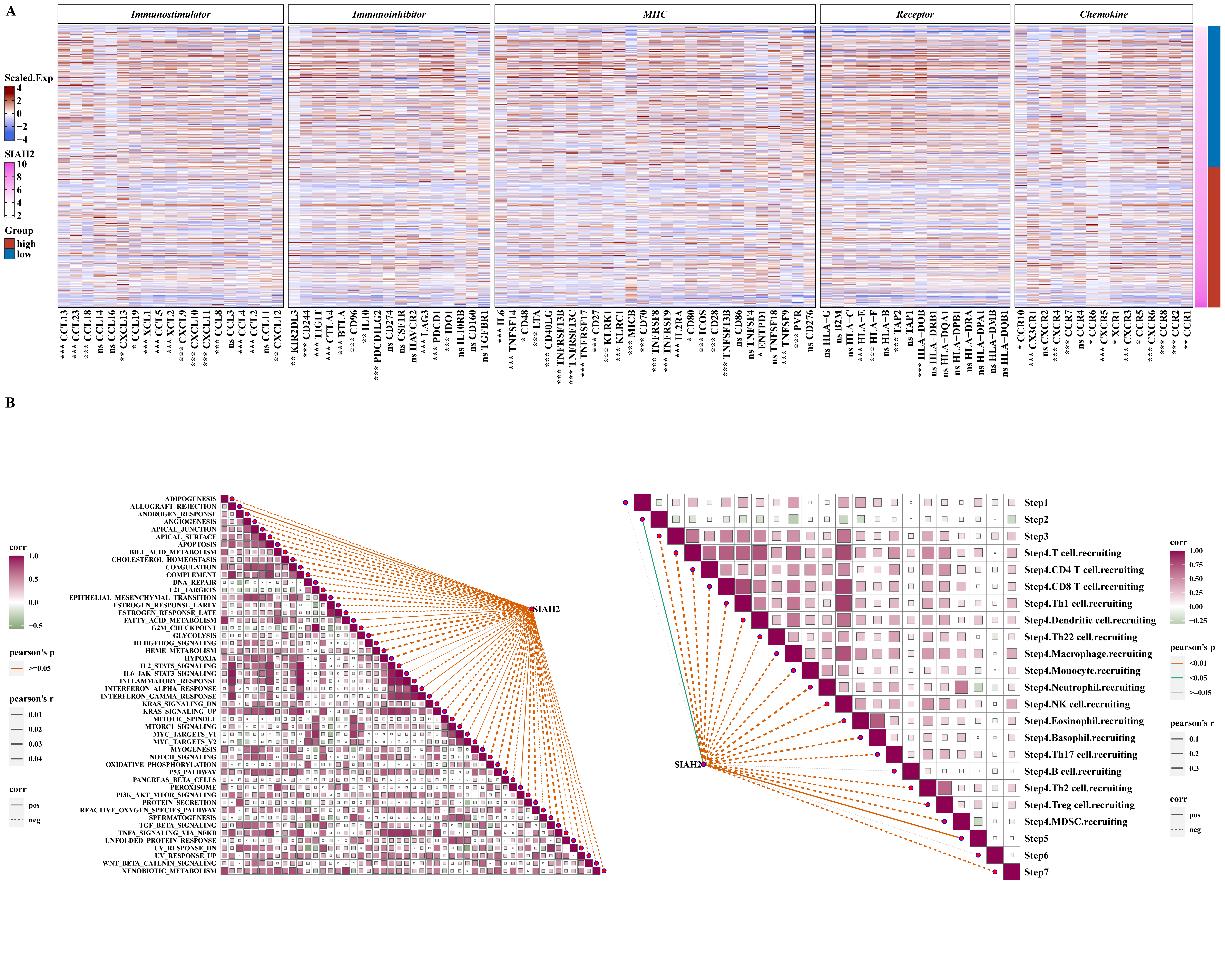

Supplement: Supplementary file 5 — Supplementary Material 5: Figure 5 A. Correlation heatmap between SIAH2 and immune checkpoint genes. B. Correlation analysis between SIAH2 and 50 HALLMARK pathways plus TIP (IMMUNO-ONCOLOGY) scores. [file 12672_2026_4528_MOESM5_ESM.tif]
